# The Immunomodulatory Role of Microbiota in Rheumatic Heart Disease: What Do We Know and What Can We Learn from Other Rheumatic Diseases?

**DOI:** 10.3390/medicina59091629

**Published:** 2023-09-08

**Authors:** Amira Kohil, Wafa Abdalla, Wisam N. Ibrahim, Khalid M. Al-Harbi, Amal Al-Haidose, Maha Al-Asmakh, Atiyeh M. Abdallah

**Affiliations:** 1 Division of Biological and Biomedical Sciences, College of Health and Life Sciences, Hamad Bin Khalifa University, Doha 34110, Qatar; 2 Department of Biomedical Sciences, College of Health Sciences, QU-Health, Qatar University, Doha 2713, Qatarmaha.alasmakh@qu.edu.qa (M.A.-A.); 3 Department of Pediatrics, College of Medicine, Taibah University, Al-Madinah 41491, Saudi Arabia; 4 Biomedical Research Center, Qatar University, Doha 2713, Qatar

**Keywords:** rheumatic heart disease, microbiota, immunomodulation, autoimmunity, molecular mimicry, epitope spreading, bystander activation

## Abstract

Rheumatic heart disease (RHD) represents a serious cardiac sequela of acute rheumatic fever, occurring in 30–45% of patients. RHD is multifactorial, with a strong familial predisposition and known environmental risk factors that drive loss of immunological tolerance. The gut and oral microbiome have recently been implicated in the pathogenesis of RHD. Disruption of the delicate balance of the microbiome, or dysbiosis, is thought to lead to autoimmune responses through several different mechanisms including molecular mimicry, epitope spreading, and bystander activation. However, data on the microbiomes of RHD patients are scarce. Therefore, in this comprehensive review, we explore the various dimensions of the intricate relationship between the microbiome and the immune system in RHD and other rheumatic diseases to explore the potential effect of microbiota on RHD and opportunities for diagnosis and treatment.

## 1. Introduction

### 1.1. Rheumatic Heart Disease

Rheumatic heart disease (RHD) is a serious cardiac sequela of acute rheumatic fever that can result in cardiac failure and valvular damage in children and young adults [1]. Rheumatic fever remains a significant healthcare burden in low- and middle-income countries [2]. Pharyngeal infection with beta-hemolytic group A streptococcal bacteria causes a systemic inflammatory response 2–3 weeks after the infection, which subsequently damages the tissues of several organs, especially the heart, joints, brain, and skin [3]. The clinical presentation is variable, but the most common manifestations are painful joints and cardiac involvement, the latter including carditis, valvular lesions, conductive system disorders, and pericardial disease. Cutaneous (erythema marginata, subcutaneous nodules) and neurological (Sydenham’s chorea) manifestations also occur.

**Figure 1:**
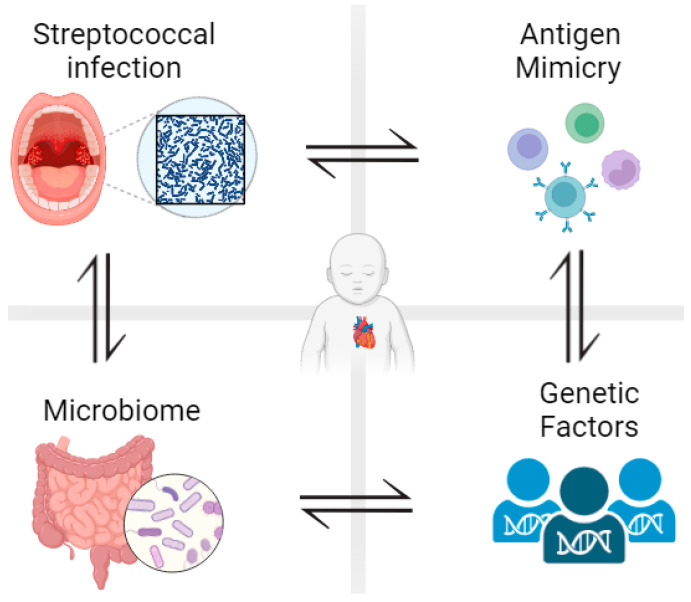
Development of rheumatic heart disease is influenced by different factors. After pharyngeal infection with beta-hemolytic group A streptococcal bacteria, less than 3% of patients develop rheumatic fever and around 40% of those patients develop rheumatic heart disease. Antigenic mimicry, genetic predisposition, and gut/oral microbiota all determine susceptibility to RHD. Figure generated using Biorender.com (accessed on 1 June 2023).

Acute rheumatic fever occurs in about 0.3–3% of people infected with group A streptococci. The inflammatory tissue damage usually resolves within weeks to months and valvular involvement in this phase is most often minimal to moderate. Nevertheless, about 30–45% of patients with acute rheumatic fever will develop permanent valvular damage and chronic rheumatic disease, in which there is persistent inflammation of heart tissue in the absence of bacteria [4,5]. This immunological reaction and the vulnerability of the infected individual to rheumatic fever or long-term RHD depend on three main factors: the bacterial strain, host genetic predisposition, and aberrant host immune responses (Figure 1) [6,7]. The virulence of GAS strains plays a pivotal role in initiating and perpetuating the cascade of events leading to RHD. Virulence factors such as M-proteins, which facilitate immune evasion, and streptolysin O, which induces tissue damage, contribute to the heightened pathogenicity of certain GAS strains. Additionally, GAS strains capable of forming biofilms on cardiac surfaces create a conducive environment for persistent infections and exacerbate immune responses. The interplay between these bacterial attributes, the host’s genetic susceptibility, and the subsequent immune responses forms the basis of RHD pathogenesis. It is important to highlight that there are several infections that may be linked to varying degrees of cardiac manifestations. For instance, *Mycoplasma pneumoniae*, as part of a spectrum of extra-pulmonary reactive manifestations, can also give rise to cardiac issues [8,9].

In terms of an underlying mechanism, RHD is thought to be driven by an autoimmune response occurring due to antigenic mimicry between specific bacterial surface proteins and antigens expressed at the cell surface of genetically predisposed individuals [10]. These bacterial surface proteins, which mimic elements of human cardiac myosin including the carbohydrate epitope, N-acetyl glucosamine, and spiral M protein, activate CD4^+^ T cells, B cells, and macrophages, which target host cells [4]. Moreover, monocyte activation produces proinflammatory cytokines including tissue necrosis factor alpha (TNF) and interleukins 1 and 2 (IL-1 and IL-2), which contribute to the long-term effects of rheumatic fever [11].

Mediated by immune activation, antigen-antibody complexes cause the degeneration of connective tissue, tissue edema, and formation of Aschoff nodules, which consist of plasma cells, macrophages, polymorphonuclear leukocytes, Anitschkow cells, multinucleated cells, and a few lymphocytes. These nodules are found in the endocardium, subendocardium, and around myocardial blood vessels [12]. Furthermore, vascular cell adhesion molecule 1 (VCAM-1) is expressed on the endocardium, which helps CD4^+^ cells to adhere to the endocardium to cause valve inflammation and neovascularization, in turn promoting blood flow and further delivery of inflammatory cells that further damage the valves [12].

A genetic predisposition for RHD is well established. Epidemiological studies have shown that individuals with parents who developed RHD are more susceptible to developing the disease, and there is 44% concordance in RHD for monozygotic twins and 12% for dizygotic twins [13], equating to a heritability of approximately 0.6 [14]. Furthermore, genome-wide association studies (GWAS) have established that certain genetic variants in the major histocompatibility complex, especially human leukocyte antigen class II, and in other immune system genes are associated with the disease [15]. However, a more recently established risk factor for RHD is dysbiosis of the oral cavity and the gut microbiota, as discussed below [16].

### 1.2. Signaling Pathway in RHD

Several signaling pathways are now known to be important in the pathogenesis of RHD. One of the most important signaling pathways involved in RHD is the Toll signaling pathway, where each member of the Toll-like receptor family recognizes distinct pathogen-associated molecular patterns (PAMPs) derived from microbial pathogens. TLR2 and TLR4 are of particular relevance to RHD, as they recognize components of group A *Streptococcus*, lipoteichoic acid and lipopolysaccharide, respectively [17]. Activation of TLR2 and TLR4 triggers a cascade of intracellular events that lead to the production of pro-inflammatory cytokines and immune cell recruitment. Most Toll-like receptors, including TLR2 and TLR4, activate the MyD88-dependent pathway, which initiates critical downstream signaling events [18], including the activation of the nuclear factor-kappa B (NF-κB) transcription factor and mitogen-activated protein kinases (MAPKs). These pathways in turn activate expression of pro-inflammatory cytokines such as IL-1β, IL-6, and tumor necrosis factor-alpha (TNF-α) [19]. Indeed, MAPK cascades including extracellular signal-regulated kinases (ERKs), c-Jun N-terminal kinases (JNKs), and p38 MAPK are activated in RHD, thereby contributing to valve degeneration and fibrosis [20]. Activation of transforming growth factor-beta (TGF-β)—a multifunctional cytokine that regulates various cellular processes including immune responses, cell proliferation, and extracellular matrix (ECM) remodeling—has also been reported in RHD, where it contributes to valvular inflammation, fibrosis, and calcification. Dysregulated TGF-β pathway activation leads to an imbalance between ECM synthesis and degradation, resulting in valvular dysfunction [21].

The lectin pathway is one of the three complement activation pathways, along with the classical and alternative pathways. The lectin pathway is initiated by the binding of pattern recognition molecules known as lectins to carbohydrate structures present on microbial surfaces. In RHD, the mannose-binding lectin (MBL) is of particular importance, since it recognizes carbohydrate moieties on the surface of group A *Streptococcus*, triggering complement activation. Dysregulated MBL-mediated lectin signaling in RHD contributes to excessive inflammation and tissue injury [22]. Complement activation plays a critical role in host defenses and immune regulation and, in RHD, it is activated via multiple mechanisms, including the lectin pathway. Complement components such as C3 and C5 contribute to the amplification of the inflammatory response by generating anaphylatoxins (C3a and C5a) and assembling the membrane attack complex (MAC), resulting in tissue injury [23]. Additionally, complement activation promotes the recruitment and activation of immune cells, exacerbating the inflammatory cascade and sustaining inflammation and tissue injury in RHD patients. Interestingly, there is emerging evidence of a bidirectional relationship between the microbiome and complement activation, wherein the microbiome influences complement activity and complement components shape the composition and function of the microbiome [24].

Therefore, our objective in this review is to understand the various dimensions of the intricate relationship between the microbiome and the immune system in RHD and other rheumatic diseases to explore the potential effect of microbiota on RHD and opportunities for diagnosis and treatment. This may help in developing prophylaxis or treatment protocols to protect children from developing RHD and permanent damage of the heart valves.

## 2. Human Microbiota

The human microbiota consists of microorganisms (bacteria, fungi, and viruses) that reside on or within the human body [25]. Different factors influence microbial composition such as diet, age, and xenobiotics, which result in large inter/intra-personal diversity in the human microbiota. In addition, the composition of microbial communities differs at different body sites (such as the gut, skin, oral cavity, and vagina) [26]. The human microbiota has been shown to play an important role in different physiological processes, such as immune homeostasis, inflammation [27], and nutrient and drug metabolism [28].

The gut microbiota is a dynamic and diverse microbial community that resides in the digestive system, and it is the largest compared with other body sites [29]. The intestinal microbiota contains over 1500 species, mainly of the phyla Bacteroidetes and Firmicutes, which account for 90% of the gut microbial community [30]. The gut microbiota plays an important role in protecting the host against pathogens by colonizing mucosal surfaces and producing microbial metabolites [31]. It also plays an essential role in digestion and metabolism. Perhaps unsurprisingly, given its physiological relevance, the human microbiome is now implicated in the development of several different diseases including cardiovascular disorders [32], metabolic diseases [33], and neurological disorders [34].

The second most complex microbial community is found in the oral cavity, and it has been shown to influence both oral and systematic health [35]. The oral microbiota is diverse, comprising ~700 species, of which only 54% have been cultured and named [36]. The oral microbial community can be altered by the presence of periodontal pathogens, which then affect microbiome–host interactions, resulting in local inflammation and periodontal tissue destruction [37]. Oral microbial dysbiosis has also been associated with different diseases, such as cancer, inflammatory bowel disease, cardiovascular disorders, Alzheimer’s disease, celiac disease, and IgA nephropathy [38,39,40]. Therefore, an in-depth understanding of the involvement of the human microbiota in health and its interaction with the host could help in the development of new therapies that maintain host-microbiota homeostasis [41]. To achieve this, it is first necessary to understand the complex interactions occurring between microbiota and host as well as microbe–microbe interactions within the host.

## 3. The Role of Microbiota in the Pathogenesis of RHD

**Figure 2:**
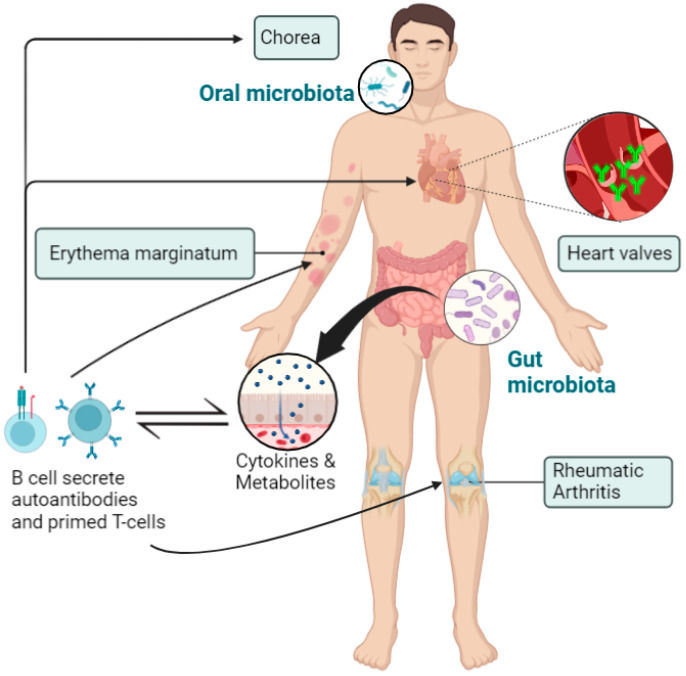
Disruption of the delicate balance of the microbiome, or dysbiosis, leads to autoimmune reactions causing a systemic inflammatory response. This immunological reaction causes damage to different tissues and organs, mainly the heart, joints, brain, and skin. Figure generated using Biorender.com (accessed on 1 June 2023).

Several studies have now implicated the gut microbiome in a number of cardiovascular disorders including pulmonary arterial hypertension [42], chronic heart failure [43], intracranial aneurysms [44], and atherosclerosis [45]. Furthermore, the oral microbiota has also been associated with cardiovascular disorders, as periodontal bacteria DNA has been detected in cardiac tissues and atherosclerotic plaques [45,46]. In addition, periodontitis (inflammatory oral disease) is associated with an increased risk of developing stroke, atherosclerosis, rheumatoid arthritis, and diabetes [47]. However, there are only limited data on the potential role of the gut and oral microbiota in the pathogenesis of RHD (Figure 2).

Shi and colleagues recently analyzed the microbial composition of RHD patients and identified a potential role for microbiota in the pathogenesis of RHD [16]. The authors showed that RHD patients have altered gut and oral microbiota, which they suggested might translocate to the mitral valves and increase the severity of the disease. They detected an increased relative abundance of *Bifidobacterium* and *Eubacterium* and decreased relative abundance of *Faecalibacterum* and *Bacteroides* in RHD patients compared with controls. Although *Bifidobacterium* and *Eubacterium* are usually considered part of the normal flora [48], several studies have detected increased levels of these organisms in patients with different inflammatory and autoimmune disorders, such as inflammatory bowel disease and systemic lupus erythematosus (SLE) [49,50]. In addition, *Faecalibacterum* and *Bacteroides* are known to be important producers of propionate and butyrate [51], which play essential roles in mediating immunity [52]. Therefore, decreased levels of *Faecalibacterum* and *Bacteroides* might decrease propionate and butyrate levels, thereby negatively impacting the immune system and promoting the development of RHD. It has also been suggested that, during progression of RHD, a decrease in some beneficial microbial genera (*Faecalibacterum* and *Bacteroides*) disrupts immune homeostasis, thus, increasing *Bifidobacterium* and *Eubacterium* as a feedback mechanism [16]. Short chain fatty acids (SCFAs), such as butyrate, produced from various gut microbial species are well known to affect immune homeostasis and inflammatory response [53]. Studies have shown that SCFAs play a role in B-cell differentiation through histone deacetylase (HDAC) inhibition [54]. Also, several studies have showed the implication of B-cells in the pathogenesis of RHD through its interaction with T-cells and enhancing the production of interleukins [55]. Therefore, reduced levels of SCFAs and its metabolite negatively impacting the immune system could be implicated in the pathogenesis of RHD. 

However, the exact function of these gut microbial genera in RHD has yet to be established. In comparison to other cardiovascular disorders, patients with chronic heart failure have a low abundance of *Bifidobacterium* [43], which is opposite to the pattern seen in RHD patients. In patients with pulmonary arterial hypertension, *Bacteroides* are decreased while *Bifidobacterium* are increased [42], similar to RHD cases [16]. However, pulmonary arterial hypertension patients have decreased abundance of *Eubacterium* [42], which were found to be increased in RHD patients [16]. Therefore, it has been proposed that the same bacteria show pleiotropism, thereby playing different roles in different cardiovascular conditions. Gong and colleagues studied a cohort of RHD patients with atrial fibrillation to examine associations between serum trimethylamine N-oxide (TMAO) (a gut microbiome metabolite) levels and thrombus formation [56], and patients who developed thrombus had higher levels of TMAO, betaine, and choline compared with patients who did not develop thrombus. In addition, TMAO levels were positively associated with platelet hyperactivity and thrombus formation in RHD patients with atrial fibrillation [56].

**Figure 3:**
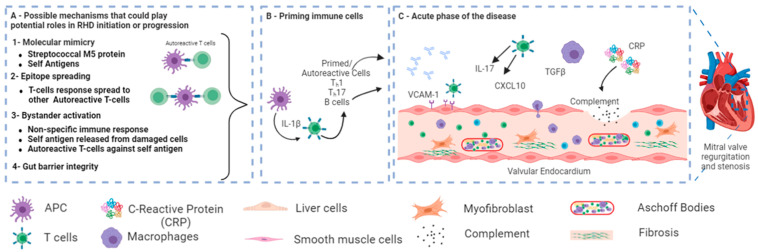
A schematic showing the possible role of microbiota in the pathogenesis of RHD after group A streptococcal (GAS) infection. (**A**) In genetically predisposed individuals, different possible mechanisms could play potential roles in RHD initiation or progression, such as molecular Mimicry between Streptococcal M protein and cardiac myosin [52] and epitope spreading [53]. Other potential mechanisms, such as bystander activation and gut barrier integrity, have not been directly linked to RHD. (**B**) Primed autoreactive cells could activate other cells. (**C**) Primed autoreactive cells, autoantibodies, complement factors, and other immunomodulators transfer to the heart and cause valvular damage. Figure reprinted/adapted with permission from refs. Abdallah et al., 2021 [6], and Carlus et al., 2020 [52].

In RHD patients, there appears to be greater microbial richness in the salivary microbiota compared with healthy controls, which may be due to poor oral hygiene [16,57]. Furthermore, *Streptococcus* levels were found to be higher in the saliva and subgingival plaques of RHD patients [16]. In the same study, *Roseburia*, *Lachnoanaerobaculum*, and *Corynebacterium* in subgingival plaques correlated positively with RHD severity. In addition, potential transmission of the oral and gut microbiome to the mitral valves in RHD patients was inferred through shared microbial genera at the different sites [16]: *Streptococcus*, *Shigella*, *Lactobacillus*, and *Bacteroides* were common in the mitral valve and fecal samples, while *Streptococcus* and *Fusobacterium* were shared between the mitral valves and saliva/subgingival plaques. In addition, increased and decreased levels of Proteobacteria and Firmicutes, respectively, were detected in the mitral valves compared with the gut, subgingival plaques, and saliva. *Streptococcus* was abundantly distributed in the mitral valves of all RHD patients, which could support the theory that streptococcal infection can translocate to the circulation and access the subendothelial collagen matrix [58]. Therefore, translocation of these microbial genera to heart valves might release antigens that induce an autoimmune response against the cardiac tissues in RHD patients (Figure 3). In addition, the blood microbiome might represent an independent biomarker for cardiovascular disorders and autoimmune rheumatic disorders [59], but there has yet to be a direct study of the blood microbiome in RHD patients.

## 4. Lessons from Other Rheumatic Disorders—The Role of Microbiota

### 4.1. Joint Involvement and Rheumatoid Arthritis

Rheumatoid arthritis (RA) is an autoimmune disease that affects the joints and is characterized by the presence of circulating rheumatoid factor and anti-citrullinate peptide antibodies. RA has a multifactorial pathogenesis, with both genetic and environmental factors involved [60]. There have now been several studies on the potential role of microbiota, mainly the oral microbiota, in the disease [61,62,63,64]. The gut is implicated in the pathogenesis of RA through intestinal epithelial barrier integrity loss and the development of gut inflammation. Matie and colleagues showed that the gut permeability of patients with active RA was enhanced compared with controls, and the permeability was positively correlated with disease severity [65]. This enhanced gut permeability in RA could be due to gut microbial dysbiosis characterized by reduced gut microbial diversity and enhanced abundance of *Eggerthella*, *Actinomyces*, *Turicibacter*, and *Streptococcus* [66]. It has also been suggested that *Actinobacteria*, mainly *Collinsella* and *Eggerthella* genera, play an important role in the pathogenesis of RA and can be used as a biomarker to predict RA status [66]. *Collinsella* might contribute to RA pathogenesis through tight junction disruption and molecular mimicry, as it shares sequences with DRB1*0401 [67], thereby leading to increased gut permeability and enhanced immune activation and inflammation. In addition, treatment with methotrexate (the main drug used in patients with RA) partially restored gut microbial dysbiosis in RA patients [62,67], further suggesting that the gut microbiota may be a useful prognostic tool. Moreover, a recent systematic review showed that the gut microbial profile of RA patients shows decreased abundance of *Faecalibacterium* and increased abundance of *Streptococcus* [61]. Another study reported increased enrichment of butyrate-consuming species and decreased enrichment of butyrate-producing bacteria in RA patients; this reduction in beneficial butyrate-producing bacteria was also correlated with anti-citrullinated peptide antibody production and severity of joint deformity [32]. Other studies have detected an increased abundance of *Prevotella copri* in RA animal models and patients and a strong correlation with early stage disease [68,69]. *Prevotella copri* was associated with severe arthritis, increased Th-17 levels, and induction of IL-6 and IL-23 (Th-17-related cytokines) [68]. Therefore, intestinal *Prevotella copri* might initiate the development of RA. It is also important to mention that the patient characteristics (disease stage (early/late), disease severity) may also contribute to the different detected microbial compositions [66]. Therefore, further functional studies are needed to clearly understand the role played by the gut microbiota in RA development.

Other autoimmune arthritides have also been reported to be associated with changes in the microbiota profile, such as in ankylosing spondylitis and psoriatic arthritis [70]. A quantitative metagenomic study found that patients with ankylosing spondylitis had increased abundance of *Prevotella melanogenic* and *Prevotella copri* and decreased abundance of *Bacteroides* spp. [71]. It has been also reported that inflammasome activation is associated with gut microbial dysbiosis in ankylosing spondylitis [72], and certain enriched species might induce autoimmunity by molecular mimicry [73].

Furthermore, in a systematic review and meta-analysis, periodontitis was found to be more frequently present in RA patients, with a risk ratio of 1.13 compared with healthy controls [74]. A recent pilot study found that *Streptococcus parasanguinis* and *Actinomyces meyeri* are abundant at subgingival sites of patients with RA, while *Gemella morbillorum*, *Kingella denitrificans*, *Prevotella melaninogenica*, and *Leptotrichia* spp. were abundant in controls [63]. Another study showed that patients positive for anti-citrullinate peptide antibodies have increased abundance of *P. gingivalis* and a dysbiotic subgingival microbiome profile [64], therefore supporting the hypothesis that the oral microbiota could be an important player in RA pathogenesis. Similarly, another study found that 75% of RA patients suffer from moderate–severe periodontitis, which was associated with anti-citrullinate peptide antibody production, a disrupted subgingival microbial profile, and increased inflammatory mediators [75]. Despite the link between RA and periodontal disease reported in various studies [74,75,76], the exact relationship and mechanism have yet to be identified. However, different mechanisms have been proposed as to how the oral microbiota is involved in RA pathogenesis, namely molecular mimicry, Th-17 activation, microbial translocation, microbial profile modulation, and *P. gingivalis* peptidylarginine deiminase (PPAD) citrullination [77].

### 4.2. Skin Involvement and Dermatomyositis

Microbiota have also been implicated in the skin manifestations observed in patients with different rheumatic diseases, such as SLE and dermatomyositis. SLE is a multifactorial autoimmune disorder with common cutaneous manifestations [78]. Since the skin microbiota is considered an important contributor to the pathology of many dermatological diseases [79], it is important to discuss its potential role in autoimmune rheumatic diseases. In a study of SLE patients with cutaneous manifestations, the skin microbial diversity was reduced, with decreased abundance of *Ralstonia*, *Klebsiella*, and *Prevotella* compared with healthy controls [78]. In addition, the same study showed an increased abundance of *Firmicutes*, *Actinobacteria*, *Staphylococcus*, and *Proteobacteria* in both lesional and unaffected skin of SLE patients. *S. aureus* and *S. epidermidis* were also identified as potential skin biomarkers for the diagnosis of SLE. Another recent study found increased *Halomonas* and decreased *Pelagibacterium*, *Novosphingobium*, and *Curvibacter* in the skin lesions of SLE patients compared with healthy skin areas [80]. The gut microbiome has also been implicated in dermatomyositis (DM), an autoimmune myopathy associated with muscle weakness and skin rashes. DM patients were reported to have lower gut microbial diversity compared with healthy controls [81], and a subgroup of DM patients with interstitial lung disease (ILD)-associated MSA had decreased abundance of *Christensenellaceae* and *Ruminococcaceae*. There are only limited data on the role of the skin microbiota in rheumatoid autoimmune disorders such as SLE, and further studies are warranted to identify and elucidate the function of specific skin microbial species in the cutaneous manifestations of patients with rheumatoid disorders.

### 4.3. Neurological Involvement and Fibromyalgia Syndrome

Various studies have shown that the gut microbial profile affects different brain processes through the so-called “gut-brain axis” [82]. Therefore, the gut microbiota could be involved in the neurological manifestations of different rheumatic disorders. Fibromyalgia syndrome (FMS) is a chronic rheumatic disorder associated with cognitive deficiencies, memory, and depression [83,84], and FMS has been associated with an altered gut microbial profile, with *Parabacteroides merdae*, *Clostridium scindens*, and *Blautia hydrogenotrophica* all differentially abundant compared with healthy controls [85]. Another study revealed involvement of the gut microbiota in FMS patients with chronic widespread musculoskeletal pain. The authors of this study detected reduced microbial diversity in FMS patients with chronic pain, with *Coprococcus* significantly depleted [86]. Finally, Garcia and colleagues detected a lower abundance of *Bifidobacterium* and *Eubacterium*, which are known to be involved in the neurotransmitter metabolism in FMS cases [87]. The same study also found a reduction in glutamate and serine levels, suggesting a reduction in neurotransmitter metabolism in FMS patients.

From the available literature regarding microbial profiles in different rheumatic disorders, we can observe the potential interplay between microbiome and immunity in the pathogenesis of these diseases. Although this will provide insight into the role of the microbiome in RHD, many studies are still needed to investigate the functional mechanism correlating the microbiome with RHD pathogenesis. Since several immunological pathways are involved in the pathogenesis of RHD, it is suggested that gut and oral microbial dysbiosis negatively affect the immune system leading to a dysregulated immunity and enhanced inflammation response in RHD. In addition, due to the similar pattern of lower gut permeability and decreased SCFAs producing bacteria between RHD [16] and other rheumatic disorders [71,88], the relationship between gut microbial alteration and immunity could be a possible player in RHD. However, till this date there is no study that functionally assesses the impact of microbiome on RHD pathogenesis. Further studies are needed on gut permeability and bacterial translocation in RHD cases, as observed in other rheumatic disorders, such as rheumatoid arthritis [89]. In addition, due to the correlation between immunity and SCFAs [90], it is essential to study the circulating and fecal levels of SCFAs in RHD patients and correlate their levels with RHD clinical indices. This will provide more insight into the microbial profile of RHD patients. In addition, identifying the SCFAs profile in these patients will further give insight into the pathogenic mechanism of the disease. Furthermore, oral and gut microbial profile are affected by several genetic and environmental stimuli (Diet, medication,, etc.), thereby, it is important to take these factors into consideration while investigating RHD cases. Although 16S microbial profiling is a valuable tool for identifying the gut microbial profile, it has several limitations, such as low taxonomical resolutions and the inability to identify several microbial strains [91]. There is also a need for large-scale studies to further understand the complex interaction between microbiome, immunity, and RHD development.

## 5. Probiotics Use in Rheumatic Diseases

Probiotics are microorganisms that regulate the gut microbiota through the secretion of various metabolites (e.g., short-chain fatty acids, SCFAs) to regulate the immune system and improve health [92,93]. Although probiotics have not been investigated as a therapeutic option for patients with RHD or in cases of rheumatic fever, lessons can be learned from other autoimmune rheumatoid disorders. In SLE, long-term supplementation with probiotics has been proposed to balance the gut microbial profile, consequently reducing antibody production and inflammation and attenuating the clinical manifestations of SLE [94]. Mu et al., showed that, in an SLE mouse model, administration of probiotics (lactobacilli and *L. reuteri*) reduced endotoxemia, reduced intestinal IL-6, increased IL-10, and improved Treg–Th17 balance [95]. In addition, a recent randomized controlled trial of RA patients assessed the impact of an anti-inflammatory diet supplemented with probiotics on cardiovascular risk factors [96] and found that probiotic supplementation improved CVD risk markers, namely blood lipid profile and B100/Apo-A1 ratio [96]. Another study showed that probiotic supplementation improved the disease activity score of RA patients and decreased serum insulin and high-sensitivity C-reactive protein (hs-CRP) levels [97]. Although the impact of probiotics on gut health is well known, the molecular and biological mechanisms underlying their anti-inflammatory and immunomodulatory role in different rheumatic disorders have yet to be fully defined. It has been suggested that probiotics mediate their beneficial effects through three main actions: antimicrobial, immune response regulation, and competitive exclusion [94]. SCFAs are important mediators of these functions as they regulate the function of different immune cell types, thereby modulating the immune response and inflammation [98,99]. Probiotics were also found to regulate T helper and T reg cell function and induce immune tolerance, since they are important players in the pathology of different rheumatic disorders [100]. Additional studies are now needed to fully understand the role of probiotics in RHD, to pave the way for personalized treatment using probiotics in these patients.

## 6. Conclusions

In conclusion, exploring microbiome variations in rheumatic diseases has yielded valuable insights into the intricate interaction between the immune system and the body’s microorganisms. However, much remains undiscovered concerning RHD. Given the belief that gut dysbiosis contributes to RA by disrupting barriers and causing inflammation, it is reasonable to speculate similar mechanisms in RHD. The complexity arises from RHDs diversity, genetic and environmental influences, and the dynamic microbiome. However, leveraging advanced sequencing and innovative research, we are poised to uncover the microbiome-RHD connections. This knowledge offers potential for innovative therapies and personalized interventions.

## Data Availability

Not applicable.

## References

[1] Marijon E., Mirabel M., Celermajer D.S., Jouven X. (2012). Rheumatic Heart Disease. Lancet.

[2] Liang Y., Yu D., Lu Q., Zheng Y., Yang Y. (2023). The Rise and Fall of Acute Rheumatic Fever and Rheumatic Heart Disease: A Mini Review. Front. Cardiovasc. Med..

[3] Cunningham M.W. (2014). Rheumatic Fever, Autoimmunity and Molecular Mimicry: The Streptococcal Connection. Int. Rev. Immunol..

[4] Guilherme L., Kalil J. (2013). Rheumatic Heart Disease: Molecules Involved in Valve Tissue Inflammation Leading to the Autoimmune Process and Anti-S. Pyogenes Vaccine. Front. Immunol..

[5] Bryant P.A., Robins-Browne R., Carapetis J.R., Curtis N. (2009). Some of the People, Some of the Time: Susceptibility to Acute Rheumatic Fever. Circulation.

[6] Muhamed B., Parks T., Sliwa K. (2020). Genetics of Rheumatic Fever and Rheumatic Heart Disease. Nat. Rev. Cardiol..

[7] Abdallah A.M., Abu-Madi M. (2021). The Genetic Control of the Rheumatic Heart: Closing the Genotype-Phenotype Gap. Front. Med..

[8] Poddighe D., Comi E.V., Brambilla I., Licari A., Bruni P., Marseglia G.L. (2018). Increased Total Serum Immunoglobulin E in Children Developing Mycoplasma Pneumoniae-Related Extra-Pulmonary Diseases. Iran. J. Allergy Asthma Immunol..

[9] Gonciarz W., Tomaszewska A., Krupa A., Rechciński T., Chałubiński M., Broncel M., Chmiela M. (2022). Antibodies towards TVLLPVIFF Amino Acid Sequence of TNF Receptor Induced by Helicobacter Pylori in Patients with Coronary Heart Disease. J. Clin. Med..

[10] Guilherme L., Kalil J., Cunningham M. (2006). Molecular Mimicry in the Autoimmune Pathogenesis of Rheumatic Heart Disease. Autoimmunity.

[11] Guilherme L., Köhler K.F., Postol E., Kalil J. (2011). Genes, Autoimmunity and Pathogenesis of Rheumatic Heart Disease. Ann. Pediatr. Cardiol..

[12] Guilherme L., Cury P., Demarchi L.M.F., Coelho V., Abel L., Lopez A.P., Oshiro S.E., Aliotti S., Cunha-Neto E., Pomerantzeff P.M.A. (2004). Rheumatic Heart Disease: Proinflammatory Cytokines Play a Role in the Progression and Maintenance of Valvular Lesions. Am. J. Pathol..

[13] Engel M.E., Stander R., Vogel J., Adeyemo A.A., Mayosi B.M. (2011). Genetic Susceptibility to Acute Rheumatic Fever: A Systematic Review and Meta-Analysis of Twin Studies. PLoS ONE.

[14] Selmi C., Lu Q., Humble M.C. (2012). Heritability versus the Role of the Environment in Autoimmunity. J. Autoimmun..

[15] Muhamed B., Shaboodien G., Engel M.E. (2020). Genetic Variants in Rheumatic Fever and Rheumatic Heart Disease. Am. J. Med. Genet..

[16] Shi X.-R., Chen B.-Y., Lin W.-Z., Li Y.-L., Wang Y.-L., Liu Y., Huang J.-J., Zhang W.-W., Ma X.-X., Shao S. (2021). Microbiota in Gut, Oral Cavity, and Mitral Valves Are Associated With Rheumatic Heart Disease. Front. Cell. Infect. Microbiol..

[17] Dong H., Sun Y., Shan F., Sun Q., Yang B. (2015). Down-Regulation of MiR-101 Contributes to Rheumatic Heart Disease Through Up-Regulating TLR2. Med. Sci. Monit..

[18] Xian S., Zeng Z. (2021). Signalling Pathways Implicated in the Pathogenesis of Rheumatic Heart Disease (Review). Exp. Ther. Med..

[19] Martin W.J., Steer A.C., Smeesters P.R., Keeble J., Inouye M., Carapetis J., Wicks I.P. (2015). Post-Infectious Group A Streptococcal Autoimmune Syndromes and the Heart. Autoimmun. Rev..

[20] Zhang D., Liu X., Chen X., Gu J., Li F., Zhang W., Zheng Y. (2014). Role of the MAPKs/TGF-Β1/TRAF6 Signaling Pathway in Atrial Fibrosis of Patients with Chronic Atrial Fibrillation and Rheumatic Mitral Valve Disease. Cardiology.

[21] Zhao Z., He D., Ling F., Chu T., Huang D., Wu H., Ge J. (2020). CD4+ T Cells and TGFβ1/MAPK Signal Pathway Involved in the Valvular Hyperblastosis and Fibrosis in Patients with Rheumatic Heart Disease. Exp. Mol. Pathol..

[22] Sharma N., Wasson M., Baro L., Chaliha M.S., Toor D. (2022). Lectin Complement Pathway Components as Risk Factors for Rheumatic Heart Disease in Assam, India. Human. Gene.

[23] Beltrame M.H., Catarino S.J., Goeldner I., Boldt A.B.W., de Messias-Reason I.J. (2014). The Lectin Pathway of Complement and Rheumatic Heart Disease. Front. Pediatr..

[24] Mattos-Graner R.O., Klein M.I., Alves L.A. (2023). The Complement System as a Key Modulator of the Oral Microbiome in Health and Disease. Crit. Rev. Microbiol..

[25] Jovel J., Dieleman L.A., Kao D., Mason A.L., Wine E. (2018). The Human Gut Microbiome in Health and Disease. Metagenomics.

[26] Ursell L.K., Clemente J.C., Rideout J.R., Gevers D., Caporaso J.G., Knight R. (2012). The Interpersonal and Intrapersonal Diversity of Human-Associated Microbiota in Key Body Sites. J. Allergy Clin. Immunol..

[27] Wastyk H.C., Fragiadakis G.K., Perelman D., Dahan D., Merrill B.D., Feiqiao B.Y., Topf M., Gonzalez C.G., Van Treuren W., Han S. (2021). Gut-Microbiota-Targeted Diets Modulate Human Immune Status. Cell.

[28] Asnicar F., Berry S.E., Valdes A.M., Nguyen L.H., Piccinno G., Drew D.A., Leeming E., Gibson R., Le Roy C., Khatib H.A. (2021). Microbiome Connections with Host Metabolism and Habitual Diet from 1,098 Deeply Phenotyped Individuals. Nat. Med..

[29] Quigley E.M. (2013). Gut Bacteria in Health and Disease. Gastroenterol. Hepatol..

[30] Robles-Alonso V., Guarner F. (2013). Progress in the Knowledge of the Intestinal Human Microbiota. Nutr. Hosp..

[31] Gomaa E.Z. (2020). Human Gut Microbiota/Microbiome in Health and Diseases: A Review. Antonie Van Leeuwenhoek.

[32] Tang W.H.W., Bäckhed F., Landmesser U., Hazen S.L. (2019). Intestinal Microbiota in Cardiovascular Health and Disease: JACC State-of-the-Art Review. J. Am. Coll. Cardiol..

[33] Karlsson F.H., Tremaroli V., Nookaew I., Bergström G., Behre C.J., Fagerberg B., Nielsen J., Bäckhed F. (2013). Gut Metagenome in European Women with Normal, Impaired and Diabetic Glucose Control. Nature.

[34] Cignarella F., Cantoni C., Ghezzi L., Salter A., Dorsett Y., Chen L., Phillips D., Weinstock G.M., Fontana L., Cross A.H. (2018). Intermittent Fasting Confers Protection in CNS Autoimmunity by Altering the Gut Microbiota. Cell. Metab..

[35] Bui F.Q., Almeida-da-Silva C.L.C., Huynh B., Trinh A., Liu J., Woodward J., Asadi H., Ojcius D.M. (2019). Association between Periodontal Pathogens and Systemic Disease. Biomed. J..

[36] Zhang Y., Wang X., Li H., Ni C., Du Z., Yan F. (2018). Human Oral Microbiota and Its Modulation for Oral Health. Biomed. Pharmacother..

[37] Hajishengallis G., Darveau R.P., Curtis M.A. (2012). The Keystone-Pathogen Hypothesis. Nat. Rev. Microbiol..

[38] Read E., Curtis M.A., Neves J.F. (2021). The Role of Oral Bacteria in Inflammatory Bowel Disease. Nat. Rev. Gastroenterol. Hepatol..

[39] Tonelli A., Lumngwena E.N., Ntusi N.A.B. (2023). The Oral Microbiome in the Pathophysiology of Cardiovascular Disease. Nat. Rev. Cardiol..

[40] Poddighe D., Kushugulova A. (2021). Salivary Microbiome in Pediatric and Adult Celiac Disease. Front. Cell. Infect. Microbiol..

[41] Ezzamouri B., Shoaie S., Ledesma-Amaro R. (2021). Synergies of Systems Biology and Synthetic Biology in Human Microbiome Studies. Front. Microbiol..

[42] Kim S., Rigatto K., Gazzana M.B., Knorst M.M., Richards E.M., Pepine C.J., Raizada M.K. (2020). Altered Gut Microbiome Profile in Patients with Pulmonary Arterial Hypertension. Hypertension.

[43] Kummen M., Mayerhofer C.C., Vestad B., Broch K., Awoyemi A., Storm-Larsen C., Ueland T., Yndestad A., Hov J.R., Trøseid M. (2018). Gut Microbiota Signature in Heart Failure Defined from Profiling of 2 Independent Cohorts. J. Am. Coll. Cardiol..

[44] Li H., Xu H., Li Y., Jiang Y., Hu Y., Liu T., Tian X., Zhao X., Zhu Y., Wang S. (2020). Alterations of Gut Microbiota Contribute to the Progression of Unruptured Intracranial Aneurysms. Nat. Commun..

[45] Koren O., Spor A., Felin J., Fåk F., Stombaugh J., Tremaroli V., Behre C.J., Knight R., Fagerberg B., Ley R.E. (2011). Human Oral, Gut, and Plaque Microbiota in Patients with Atherosclerosis. Proc. Natl. Acad. Sci. USA.

[46] Ziebolz D., Jahn C., Pegel J., Semper-Pinnecke E., Mausberg R.F., Waldmann-Beushausen R., Schöndube F.A., Danner B.C. (2018). Periodontal Bacteria DNA Findings in Human Cardiac Tissue—Is There a Link of Periodontitis to Heart Valve Disease?. Int. J. Cardiol..

[47] Pihlstrom B.L., Michalowicz B.S., Johnson N.W. (2005). Periodontal Diseases. Lancet.

[48] Toscano M., De Grandi R., Stronati L., De Vecchi E., Drago L. (2017). Effect of Lactobacillus Rhamnosus HN001 and Bifidobacterium Longum BB536 on the Healthy Gut Microbiota Composition at Phyla and Species Level: A Preliminary Study. World J. Gastroenterol..

[49] Wang W., Chen L., Zhou R., Wang X., Song L., Huang S., Wang G., Xia B. (2014). Increased Proportions of Bifidobacterium and the Lactobacillus Group and Loss of Butyrate-Producing Bacteria in Inflammatory Bowel Disease. J. Clin. Microbiol..

[50] He Z., Shao T., Li H., Xie Z., Wen C. (2016). Alterations of the Gut Microbiome in Chinese Patients with Systemic Lupus Erythematosus. Gut Pathog..

[51] Blaak E.E., Canfora E.E., Theis S., Frost G., Groen A.K., Mithieux G., Nauta A., Scott K., Stahl B., van Harsselaar J. (2020). Short Chain Fatty Acids in Human Gut and Metabolic Health. Benef. Microbes.

[52] Arpaia N., Campbell C., Fan X., Dikiy S., Van Der Veeken J., Deroos P., Liu H., Cross J.R., Pfeffer K., Coffer P.J. (2013). Metabolites Produced by Commensal Bacteria Promote Peripheral Regulatory T-Cell Generation. Nature.

[53] Siddiqui M.T., Cresci G.A.M. (2021). The Immunomodulatory Functions of Butyrate. J. Inflamm. Res..

[54] Kim M., Qie Y., Park J., Kim C.H. (2016). Gut Microbial Metabolites Fuel Host Antibody Responses. Cell. Host Microbe.

[55] Liu Z., Wang Y., Li F., Xie F., Liu M., Shi J., Dong N. (2020). Circulating Follicular T Helper Cells and Humoral Reactivity in Rheumatic Heart Disease. Life Sci..

[56] Gong D., Zhang L., Zhang Y., Wang F., Zhao Z., Zhou X. (2019). Gut Microbial Metabolite Trimethylamine N-Oxide Is Related to Thrombus Formation in Atrial Fibrillation Patients. Am. J. Med. Sci..

[57] Maharaj B., Vayej A.C. (2012). Oral Health of Patients with Severe Rheumatic Heart Disease. Cardiovasc. J. Afr..

[58] Tandon R., Sharma M., Chandrashekhar Y., Kotb M., Yacoub M.H., Narula J. (2013). Revisiting the Pathogenesis of Rheumatic Fever and Carditis. Nat. Rev. Cardiol..

[59] Khan I., Khan I., Jianye Z., Xiaohua Z., Khan M., Hilal M.G., Kakakhel M.A., Mehmood A., Lizhe A., Zhiqiang L. (2022). Exploring Blood Microbial Communities and Their Influence on Human Cardiovascular Disease. J. Clin. Lab. Anal..

[60] Tobón G.J., Youinou P., Saraux A. (2010). The Environment, Geo-Epidemiology, and Autoimmune Disease: Rheumatoid Arthritis. J. Autoimmun..

[61] Chu X.-J., Cao N.-W., Zhou H.-Y., Meng X., Guo B., Zhang H.-Y., Li B.-Z. (2021). The Oral and Gut Microbiome in Rheumatoid Arthritis Patients: A Systematic Review. Rheumatology.

[62] Artacho A., Isaac S., Nayak R., Flor-Duro A., Alexander M., Koo I., Manasson J., Smith P.B., Rosenthal P., Homsi Y. (2021). The Pretreatment Gut Microbiome Is Associated with Lack of Response to Methotrexate in New-Onset Rheumatoid Arthritis. Arthritis Rheumatol..

[63] Lehenaff R., Tamashiro R., Nascimento M.M., Lee K., Jenkins R., Whitlock J., Li E.C., Sidhu G., Anderson S., Progulske-Fox A. (2021). Subgingival Microbiome of Deep and Shallow Periodontal Sites in Patients with Rheumatoid Arthritis: A Pilot Study. BMC Oral. Health.

[64] Cheng Z., Do T., Mankia K., Meade J., Hunt L., Clerehugh V., Speirs A., Tugnait A., Emery P., Devine D. (2021). Dysbiosis in the Oral Microbiomes of Anti-CCP Positive Individuals at Risk of Developing Rheumatoid Arthritis. Ann. Rheum. Dis..

[65] Matei D.E., Menon M., Alber D.G., Smith A.M., Nedjat-Shokouhi B., Fasano A., Magill L., Duhlin A., Bitoun S., Gleizes A. (2021). Intestinal Barrier Dysfunction Plays an Integral Role in Arthritis Pathology and Can Be Targeted to Ameliorate Disease. Med.

[66] Chen J., Wright K., Davis J.M., Jeraldo P., Marietta E.V., Murray J., Nelson H., Matteson E.L., Taneja V. (2016). An Expansion of Rare Lineage Intestinal Microbes Characterizes Rheumatoid Arthritis. Genome Med..

[67] Zhang X., Zhang D., Jia H., Feng Q., Wang D., Liang D., Wu X., Li J., Tang L., Li Y. (2015). The Oral and Gut Microbiomes Are Perturbed in Rheumatoid Arthritis and Partly Normalized after Treatment. Nat. Med..

[68] Maeda Y., Kurakawa T., Umemoto E., Motooka D., Ito Y., Gotoh K., Hirota K., Matsushita M., Furuta Y., Narazaki M. (2016). Dysbiosis Contributes to Arthritis Development via Activation of Autoreactive T Cells in the Intestine. Arthritis Rheumatol..

[69] Scher J.U., Sczesnak A., Longman R.S., Segata N., Ubeda C., Bielski C., Rostron T., Cerundolo V., Pamer E.G., Abramson S.B. (2013). Expansion of Intestinal Prevotella Copri Correlates with Enhanced Susceptibility to Arthritis. Elife.

[70] Gilis E., Mortier C., Venken K., Debusschere K., Vereecke L., Elewaut D. (2018). The Role of the Microbiome in Gut and Joint Inflammation in Psoriatic Arthritis and Spondyloarthritis. J. Rheumatol. Suppl..

[71] Wen C., Zheng Z., Shao T., Liu L., Xie Z., Le Chatelier E., He Z., Zhong W., Fan Y., Zhang L. (2017). Quantitative Metagenomics Reveals Unique Gut Microbiome Biomarkers in Ankylosing Spondylitis. Genome Biol..

[72] Guggino G., Mauro D., Rizzo A., Alessandro R., Raimondo S., Bergot A.S., Rahman M.A., Ellis J.J., Milling S., Lories R. (2021). Inflammasome Activation in Ankylosing Spondylitis is Associated with Gut Dysbiosis. Arthritis Rheumatol..

[73] Zhou C., Zhao H., Xiao X.Y., Chen B.D., Guo R.J., Wang Q., Chen H., Zhao L.D., Zhang C.C., Jiao Y.H. (2020). Metagenomic Profiling of the Pro-Inflammatory Gut Microbiota in Ankylosing Spondylitis. J. Autoimmun..

[74] Fuggle N.R., Smith T.O., Kaul A., Sofat N. (2016). Hand to Mouth: A Systematic Review and Meta-Analysis of the Association between Rheumatoid Arthritis and Periodontitis. Front. Immunol..

[75] Eriksson K., Fei G., Lundmark A., Benchimol D., Lee L., Hu Y.O.O., Kats A., Saevarsdottir S., Catrina A.I., Klinge B. (2019). Periodontal Health and Oral Microbiota in Patients with Rheumatoid Arthritis. J. Clin. Med..

[76] Beyer K., Zaura E., Brandt B.W., Buijs M.J., Brun J.G., Crielaard W., Bolstad A.I. (2018). Subgingival Microbiome of Rheumatoid Arthritis Patients in Relation to Their Disease Status and Periodontal Health. PLoS ONE.

[77] du Teil Espina M., Gabarrini G., Harmsen H.J.M., Westra J., van Winkelhoff A.J., van Dijl J.M. (2019). Talk to Your Gut: The Oral-Gut Microbiome Axis and Its Immunomodulatory Role in the Etiology of Rheumatoid Arthritis. FEMS Microbiol. Rev..

[78] Huang C., Yi X., Long H., Zhang G., Wu H., Zhao M., Lu Q. (2020). Disordered Cutaneous Microbiota in Systemic Lupus Erythematosus. J. Autoimmun..

[79] Zeeuwen P.L., Kleerebezem M., Timmerman H.M., Schalkwijk J. (2013). Microbiome and Skin Diseases. Curr. Opin. Allergy Clin. Immunol..

[80] Zhou H.Y., Cao N.W., Guo B., Chen W.J., Tao J.H., Chu X.J., Meng X., Zhang T.X., Li B.Z. (2021). Systemic Lupus Erythematosus Patients Have a Distinct Structural and Functional Skin Microbiota Compared with Controls. Lupus.

[81] Bae S.S., Dong T.S., Wang J., Lagishetty V., Katzka W., Jacobs J.P., Charles-Schoeman C. (2022). Altered Gut Microbiome in Patients with Dermatomyositis. ACR Open Rheumatol..

[82] Bercik P., Collins S.M. (2014). The Effects of Inflammation, Infection and Antibiotics on the Microbiota-Gut-Brain Axis. Adv. Exp. Med. Biol..

[83] Wolfe F., Smythe H.A., Yunus M.B., Bennett R.M., Bombardier C., Goldenberg D.L., Tugwell P., Campbell S.M., Abeles M., Clark P. (1990). The American College of Rheumatology 1990 Criteria for the Classification of Fibromyalgia. Report of the Multicenter Criteria Committee. Arthritis Rheum..

[84] Glass J.M. (2009). Review of Cognitive Dysfunction in Fibromyalgia: A Convergence on Working Memory and Attentional Control Impairments. Rheum. Dis. Clin. North. Am..

[85] Minerbi A., Gonzalez E., Brereton N.J.B., Anjarkouchian A., Dewar K., Fitzcharles M.A., Chevalier S., Shir Y. (2019). Altered Microbiome Composition in Individuals with Fibromyalgia. Pain.

[86] Freidin M.B., Stalteri M.A., Wells P.M., Lachance G., Baleanu A.F., Bowyer R.C.E., Kurilshikov A., Zhernakova A., Steves C.J., Williams F.M.K. (2021). An Association between Chronic Widespread Pain and the Gut Microbiome. Rheumatology.

[87] Clos-Garcia M., Andrés-Marin N., Fernández-Eulate G., Abecia L., Lavín J.L., van Liempd S., Cabrera D., Royo F., Valero A., Errazquin N. (2019). Gut Microbiome and Serum Metabolome Analyses Identify Molecular Biomarkers and Altered Glutamate Metabolism in Fibromyalgia. EBioMedicine.

[88] He J., Chu Y., Li J., Meng Q., Liu Y., Jin J., Wang Y., Wang J., Huang B., Shi L. (2022). Intestinal Butyrate-Metabolizing Species Contribute to Autoantibody Production and Bone Erosion in Rheumatoid Arthritis. Sci. Adv..

[89] Audo R., Sanchez P., Rivière B., Mielle J., Tan J., Lukas C., Macia L., Morel J., Immediato Daien C. (2022). Rheumatoid Arthritis Is Associated with Increased Gut Permeability and Bacterial Translocation Which Are Reversed by Inflammation Control. Rheumatology.

[90] Yao Y., Cai X., Fei W., Ye Y., Zhao M., Zheng C. (2022). The Role of Short-Chain Fatty Acids in Immunity, Inflammation and Metabolism. Crit. Rev. Food Sci. Nutr..

[91] Muhamad Rizal N.S., Neoh H., Ramli R., Periyasamy P.R., Hanafiah A., Abdul Samat M.N., Tan T.L., Wong K.K., Nathan S., Chieng S. (2020). Advantages and Limitations of 16S RRNA Next-Generation Sequencing for Pathogen Identification in the Diagnostic Microbiology Laboratory: Perspectives from a Middle-Income Country. Diagnostics.

[92] Bron P.A., Kleerebezem M., Brummer R.-J., Cani P.D., Mercenier A., MacDonald T.T., Garcia-Ródenas C.L., Wells J.M. (2017). Can Probiotics Modulate Human Disease by Impacting Intestinal Barrier Function?. Br. J. Nutr..

[93] de Oliveira G.L.V., Leite A.Z., Higuchi B.S., Gonzaga M.I., Mariano V.S. (2017). Intestinal Dysbiosis and Probiotic Applications in Autoimmune Diseases. Immunology.

[94] Esmaeili S., Mahmoudi M., Momtazi A.A., Sahebkar A., Doulabi H., Rastin M. (2017). Tolerogenic Probiotics: Potential Immunoregulators in Systemic Lupus Erythematosus. J. Cell. Physiol..

[95] Mu Q., Zhang H., Liao X., Lin K., Liu H., Edwards M.R., Ahmed S.A., Yuan R., Li L., Cecere T.E. (2017). Control of Lupus Nephritis by Changes of Gut Microbiota. Microbiome.

[96] Hulander E., Bärebring L., Turesson Wadell A., Gjertsson I., Calder P.C., Winkvist A., Lindqvist H.M. (2021). Diet Intervention Improves Cardiovascular Profile in Patients with Rheumatoid Arthritis: Results from the Randomized Controlled Cross-over Trial ADIRA. Nutr. J..

[97] Zamani B., Golkar H.R., Farshbaf S., Emadi-Baygi M., Tajabadi-Ebrahimi M., Jafari P., Akhavan R., Taghizadeh M., Memarzadeh M.R., Asemi Z. (2016). Clinical and Metabolic Response to Probiotic Supplementation in Patients with Rheumatoid Arthritis: A Randomized, Double-blind, Placebo-controlled Trial. Int. J. Rheum. Dis..

[98] Lee S., Koh J., Chang Y., Kim H.Y., Chung D.H. (2019). Invariant NKT Cells Functionally Link Microbiota-Induced Butyrate Production and Joint Inflammation. J. Immunol..

[99] Hui W., Yu D., Cao Z., Zhao X. (2019). Butyrate Inhibit Collagen-Induced Arthritis via Treg/IL-10/Th17 Axis. Int. Immunopharmacol..

[100] Oliviero F., Spinella P. (2020). Benefits of Probiotics in Rheumatic Diseases. Front. Nutr..

